# Supplementary material for: Inferring Selective Constraint from Population Genomic Data Suggests Recent Regulatory Turnover in the Human Brain
Source: Genome Biol Evol. 2015 Nov 19;7(12):3511–28. doi: 10.1093/gbe/evv228 (PMC4700959; doi:10.1093/gbe/evv228)
Supplement: Supplementary Data [file supp_7_12_3511__index.html]

Inferring Selective Constraint from Population Genomic Data Suggests Recent Regulatory Turnover in the Human Brain — Supplementary Data 

# Inferring Selective Constraint from Population Genomic Data Suggests Recent Regulatory Turnover in the Human Brain

## Supplementary Data

files

- Supplementary Data - zip file
