# Supplementary material for: Inferring Selective Constraint from Population Genomic Data Suggests Recent Regulatory Turnover in the Human Brain
Source: Genome Biol Evol. 2015 Nov 19;7(12):3511–28. doi: 10.1093/gbe/evv228 (PMC4700959; doi:10.1093/gbe/evv228)
Supplement: Supplementary Data [file supp_evv228_suppl_data.zip › SupplementaryTextS1.docx]

**SUPPLEMENTARY METHODS**

**Assessing classification accuracy using forward simulations**

In order to assess the accuracy of our classification approach we used SLiM ([Messer 2013](#_ENREF_4)) to perform forward simulations of 10 kilobase regions (the same window size we used for real data; see below) evolving strictly under drift, containing a region experiencing purifying selection, or experiencing positive selection across the entire region. For our simulations including purifying selection, we set the selection coefficient (2*Ns*, where *N* is the initial total population size and 1-*s* and 1-0.5*s* are the fitnesses of homozygotes and heterozygotes, respectively) to either 50, 100, or 500, and set the length of the constrained region *L* to either 2.5 kb, 5 kb, 7.5 kb, or the full 10 kb window. The start of this constrained region was always located on the left end of the simulated chromosome. Each of these simulations followed the demographic scenario reported by Tennessen *et al.* ([2012](#_ENREF_5)), which models the divergence of Europeans from the ancestral African population, and subsequent population size changes for these two populations. For these simulations we set the mutation rate to 1.2×10^-8^ mutations per base pair ([Kong et al. 2012](#_ENREF_3)) in some instances, or to reduced (6×10^-9^) or elevated (2.4×10^-8^) rates in others. Similarly, we set the recombination rate to either 1×10^-8^ crossovers per base pair (or 1 cM/Mb), or to reduced or elevated values of 1×10^-6^ or 1×10^-7^, respectively.

For each combination of 2*Ns*, *L*, mutation rate, and recombination rate, we performed 500 independent replicate simulations for training and testing our classifier. Each of these simulations was initialized from a burn-in simulation proceeding for 4,000,000 generations under equilibrium demography, using the same combination of 2*Ns*, *L*, mutation rate, and recombination rate. From each of the 500 replicate simulations we sampled 1000 chromosomes without replacement form each of the two subpopulations simulated in the Tennessen *et al.* model ([2012](#_ENREF_5)), yielding a total sample size of 2,000 individuals. We ran all burn-in and replicate simulations using a version of SLiM that we modified to simulate additive rather than multiplicative fitness effects. We then summarized the output of each simulated window by the feature vector **ξ**=[*ξ*_0_ *ξ*_1_ *ξ*_2_ … *ξ_n_*_-1_] where *n* is the number of chromosomes in the sample, *ξ_i_* is the fraction of sites in the window with a derived allele segregating at frequency *i*, except *ξ_0_*, which is the fraction of sites in the window that are monomorphic (including derived fixations). The feature vector **ξ** is thus the site frequency spectrum (SFS) modified to divide the value in each bin by the number of sites rather than the number of polymorphisms, and to include the fraction of sites that are monomorphic.

Next, for each combination of 2*Ns*, *L*, mutation, and recombination rates, we sought to train an SVM to classify simulated regions as constrained or unconstrained based on their feature vectors **ξ**. First, we constructed a training set using the output from 300 simulations with no selection and 300 windows including a region experiencing negative selection. We performed additional simulations to construct a balanced test set (200 selected and 200 unselected windows) with the same parameter combination for later use. For each combination of 2*Ns* and *L*, we constructed training and test sets were also with variable mutation rates, variable recombination rates, or both, by drawing equal numbers of simulated examples from each of the three rate values listed above. Next, we collapsed these feature vectors to contain 1,000 bins as we found that this amount of binning improved cross-validation accuracy on our training set made from real genomic data (see below). We then formatted feature vectors for these training and test sets for use by LIBSVM ([Chang and Lin 2011](#_ENREF_1)), and rescaled them using the svm-scale command with default scaling parameters; the training and test sets were concatenated together for this step (along with positively selected simulations; see below) to ensure the same scaling parameters were used for both sets.

We then used LIBSVM’s svm-train command with a radial basis kernel function to learn the hyperplane optimally separating the conserved and unconserved training data according to the SVM’s *C* parameter ([Cortes and Vapnik 1995](#_ENREF_2)). The hyperplane chosen, and therefore its accuracy when classifying data not included in the training set, depend on this *C* parameter and the radial basis function’s *γ* parameter. We therefore performed a grid search of these two parameter values, examining all powers of two between 2^-11^ and 2^9^ for each parameter. For each combination of *C* and *γ*, we performed 10-fold cross validation in order to assess the SVM’s accuracy. We then used the optimal combination of hyperparameters to train an SVM from the entire training set, and assessed the accuracy of this SVM using the test set.

In some cases, the optimal combination of the *C* and gamma *γ* yielded poor accuracy on the test set only when using LIBSVM’s option to compute posterior classification probabilities: in such cases the classification probability for the feature vector being classified was nearly always exactly 0.5 for both classes. We only observed this behavior when one or both of the *C* or *γ* hyperparameters was very small (i.e. 2^-6^ or less). We therefore slightly modified our grid search procedure to obtain all hyperparameter combinations with a cross-validation accuracy value within 1% of that of the optimal combination, and then selected from these the combination with the smallest sum of |log_2_*C*|+|log_2_*λ*|, thereby punishing hyperparameters whose base-2 exponent differed greatly from zero. After this modification, all grid searches produced SVMs that had similar performance on the test set as on the training set and emitted a more continuous range of probability estimates. Crucially, the poor posterior probability estimation described above was not exhibited by the final SVM learned from the 1000 Genomes data (as described below), which produced a more uniform range of probability estimates and whose performance we also assessed using independent test sets.

We also simulated a set of 400 population samples experiencing recurrent positive selection with each mutation at each site in the chromosome being positively selected with 2*Ns*=100 (where homozygote and heterozygote fitness values are 1+*s* and 1+0.5*s*, respectively). We then asked what fraction of these positively selected samples were classified as negatively selected or unselected by each SVM. For these simulations, the mutation rate was set to 1.2×10^-8^ and the recombination rate to 1×10^-8^.

**SUPPLEMENTARY REFERENCES**

Chang C-C and Lin C-J. 2011. LIBSVM: a library for support vector machines. ACM Transactions on Intelligent Systems and Technology (TIST) 2: 27.

Cortes C and Vapnik V. 1995. Support-vector networks. Machine learning 20: 273-297.

Kong A, Frigge ML, Masson G, et al. 2012. Rate of de novo mutations and the importance of father/'s age to disease risk. Nature 488: 471-475.

Messer PW. 2013. SLiM: simulating evolution with selection and linkage. Genetics 194: 1037-1039.

Tennessen JA, Bigham AW, O’Connor TD, et al. 2012. Evolution and functional impact of rare coding variation from deep sequencing of human exomes. science 337: 64-69.
