# Supplementary figures and images for: Inferring Selective Constraint from Population Genomic Data Suggests Recent Regulatory Turnover in the Human Brain
Source: Genome Biol Evol. 2015 Nov 19;7(12):3511–28. doi: 10.1093/gbe/evv228 (PMC4700959; doi:10.1093/gbe/evv228)

Figure S1

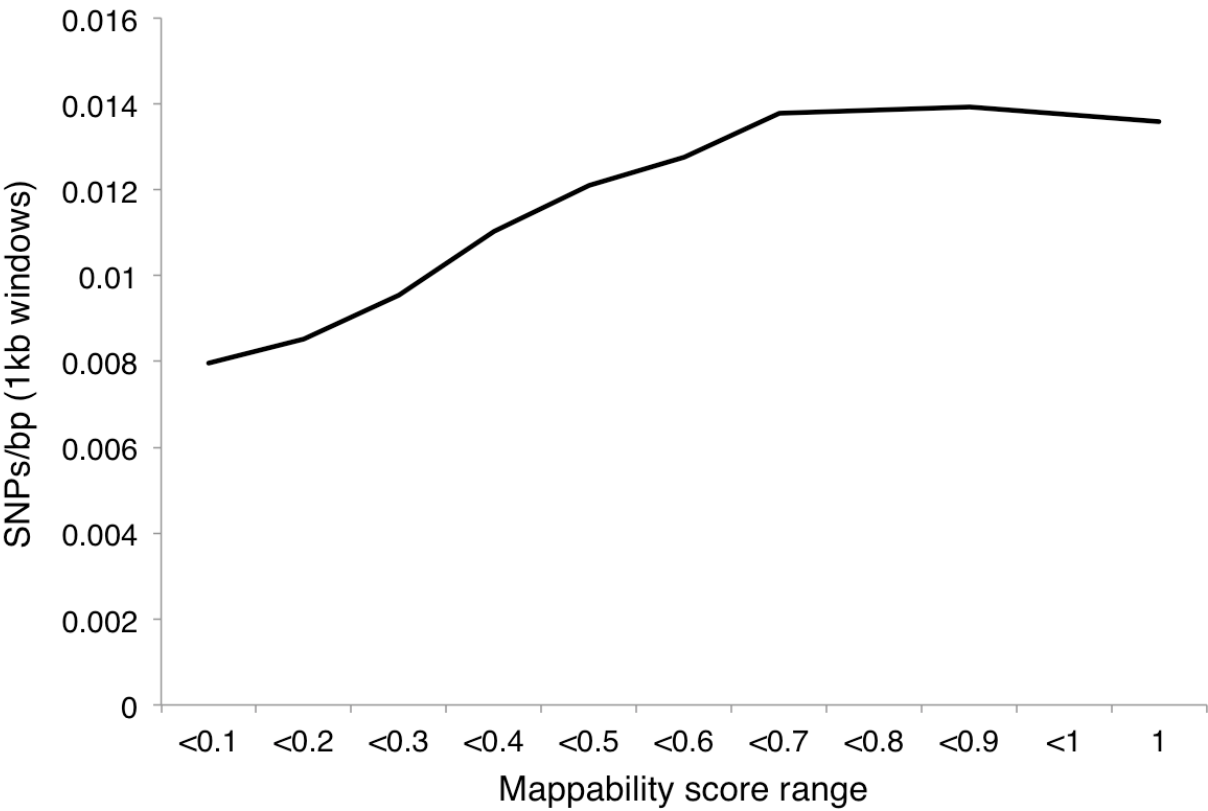

Supplement: Supplementary Data [file supp_evv228_suppl_data.zip › Figure_S1.pdf]

Figure S2

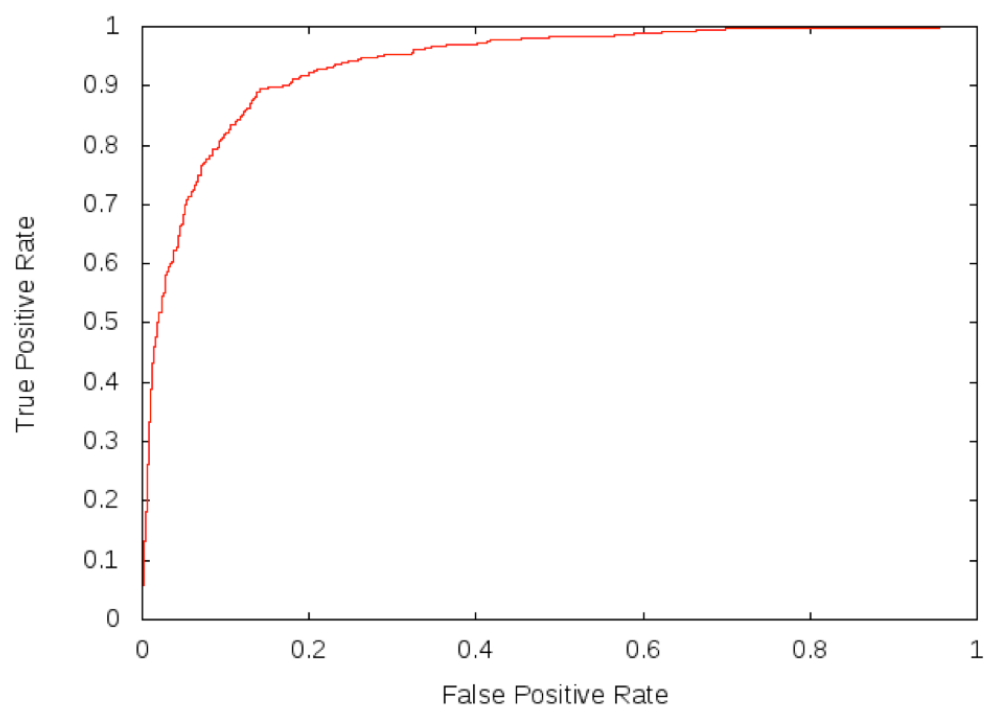

Supplement: Supplementary Data [file supp_evv228_suppl_data.zip › Figure_S2.pdf]

Figure S3

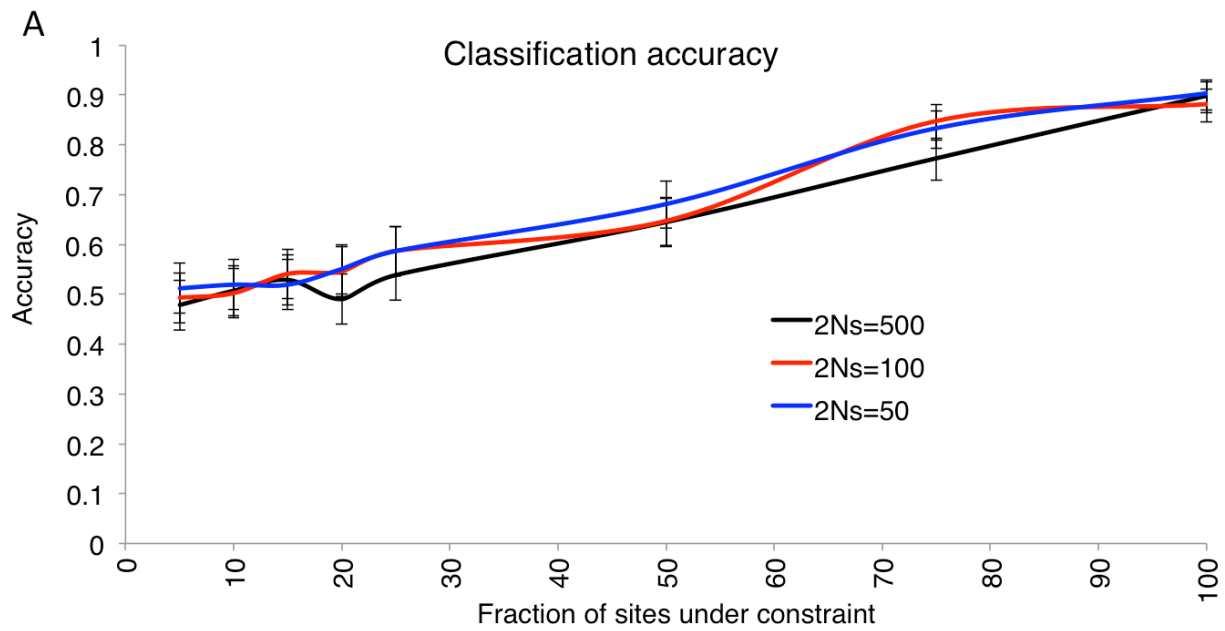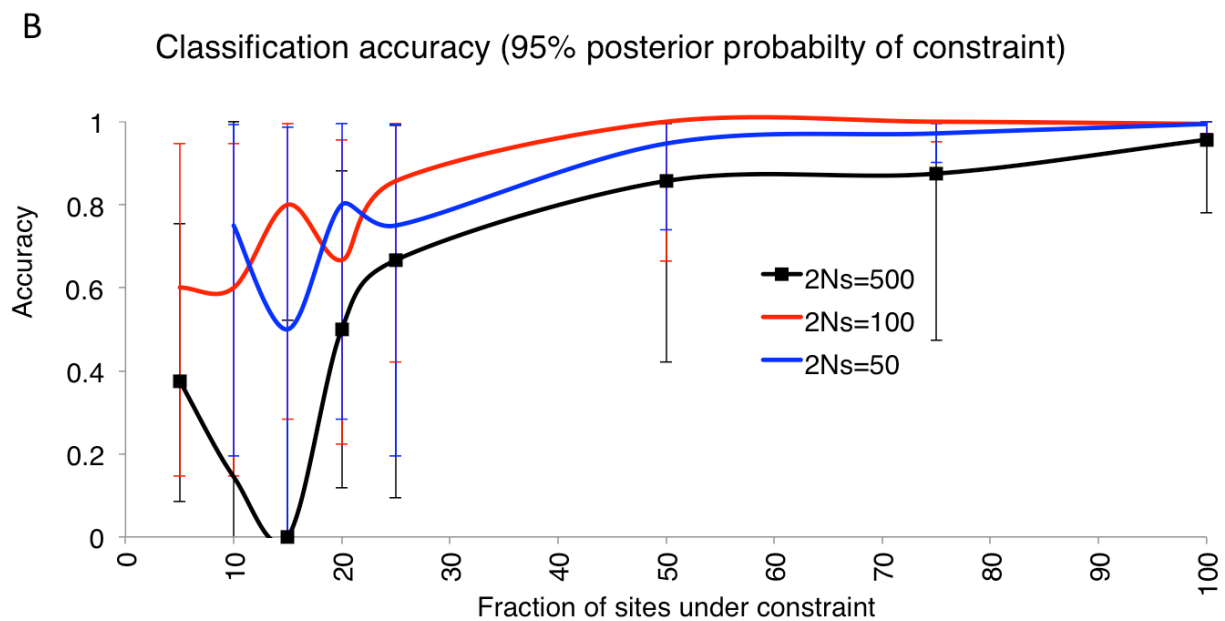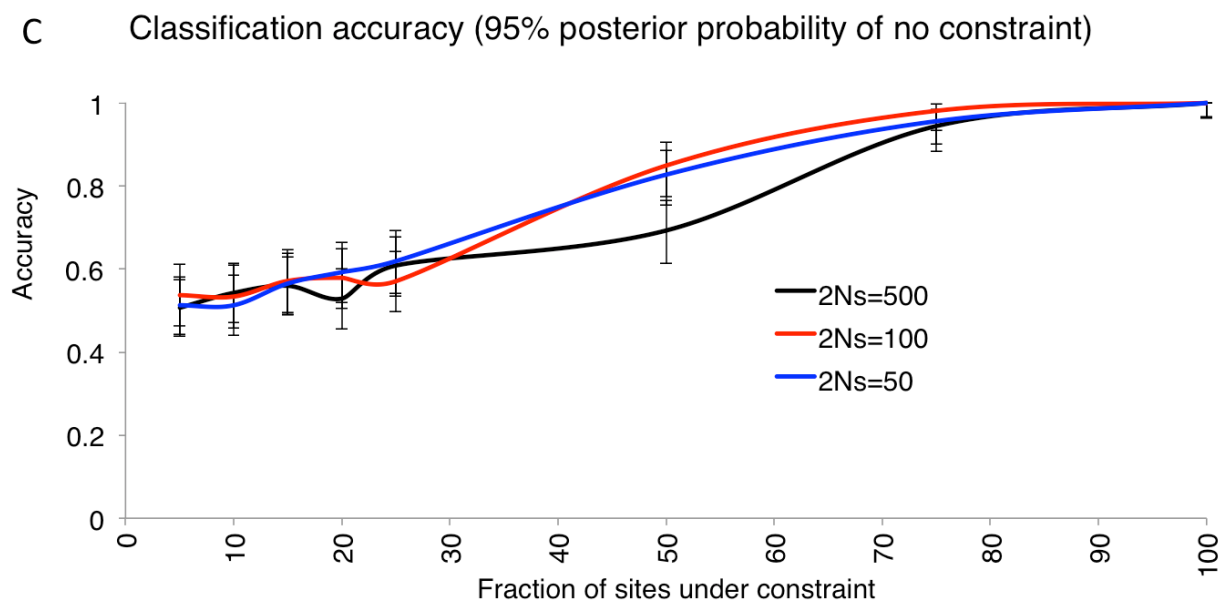

Supplement: Supplementary Data [file supp_evv228_suppl_data.zip › Figure_S3.pdf]

Figure S4

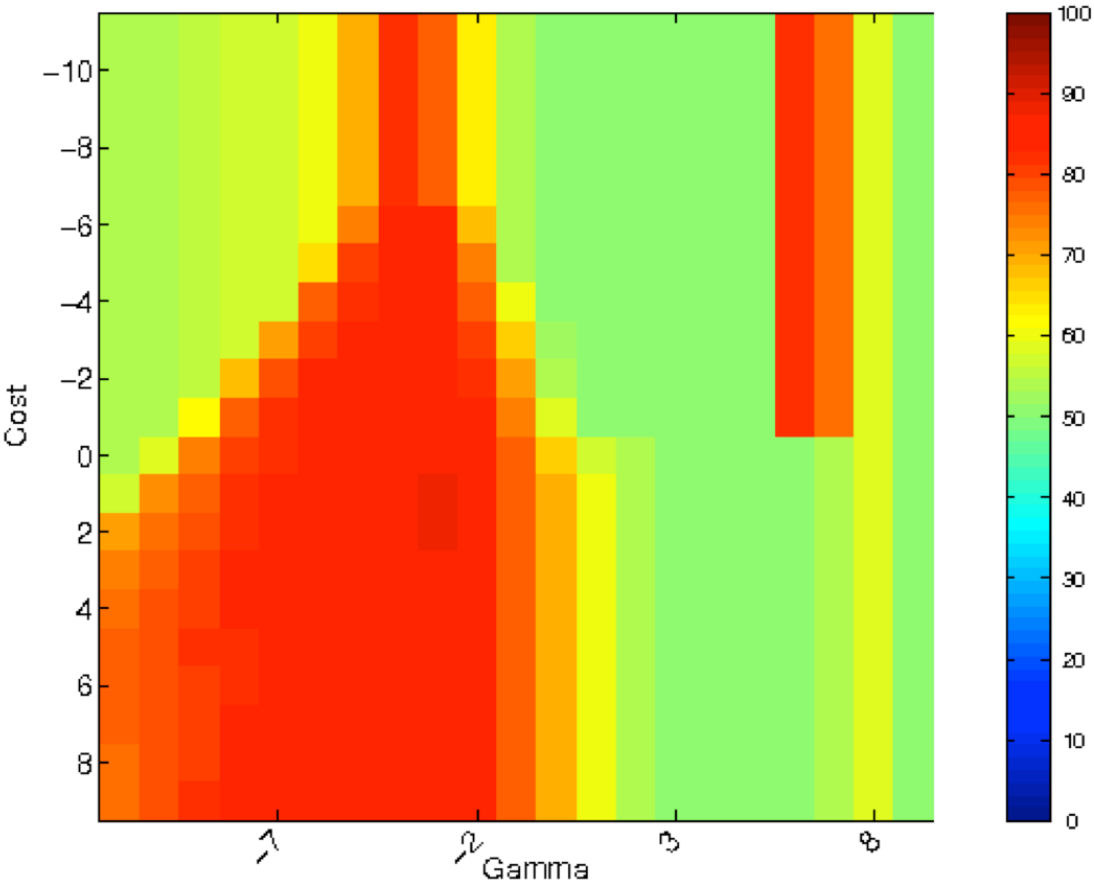

Supplement: Supplementary Data [file supp_evv228_suppl_data.zip › Figure_S4.pdf]

Figure S5

A

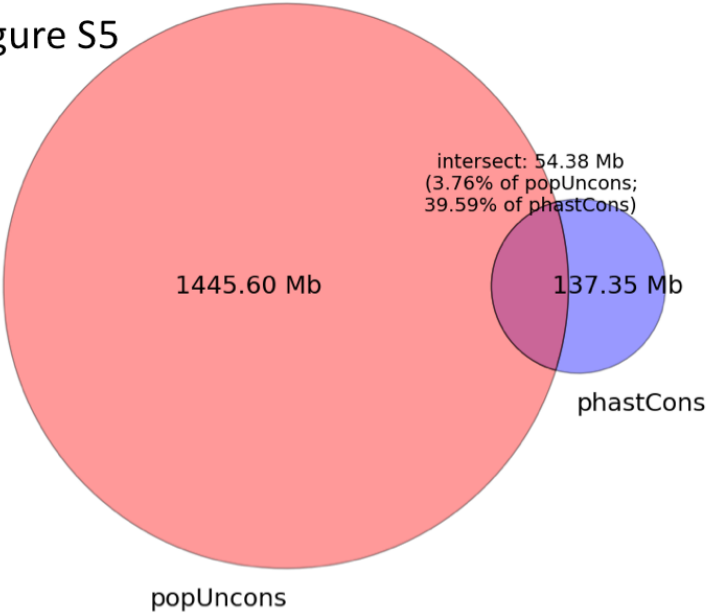

B

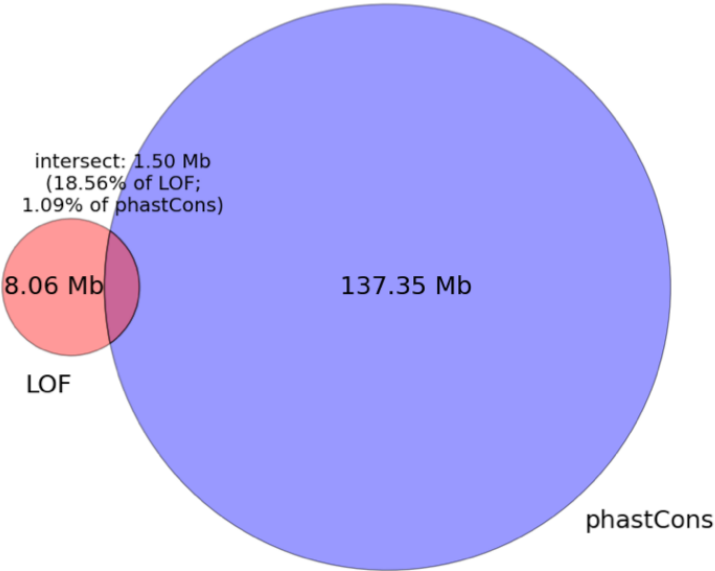

C

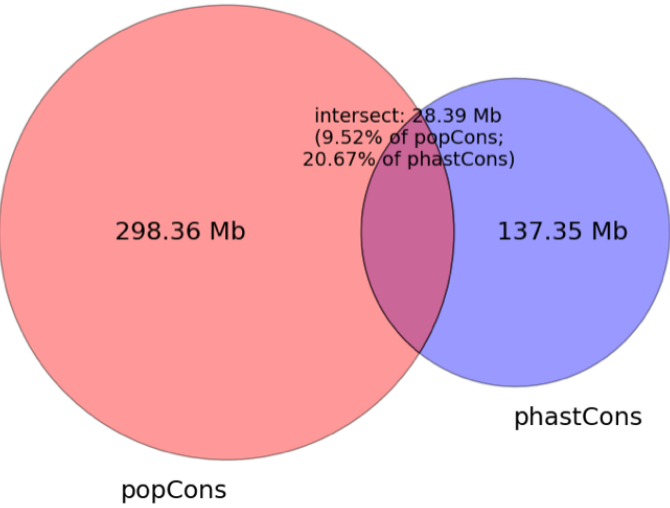

D

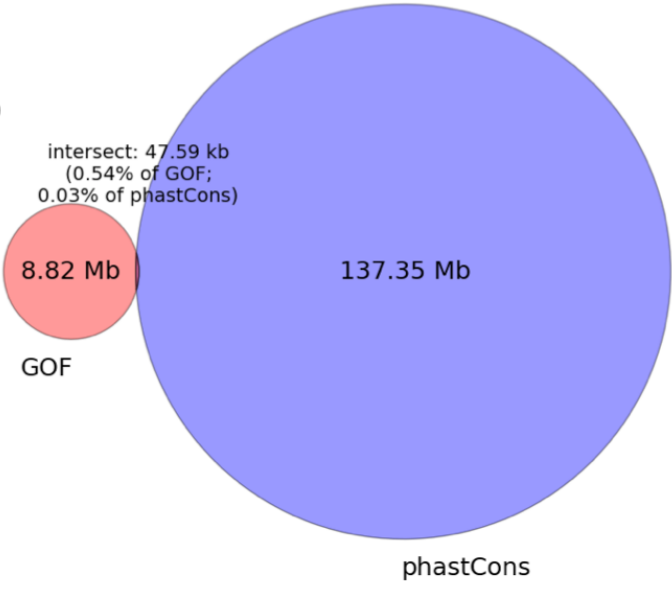

Supplement: Supplementary Data [file supp_evv228_suppl_data.zip › Figure_S5.pdf]
